# The role of cholesterol in invasion and growth of malaria parasites

**DOI:** 10.3389/fcimb.2022.984049

**Published:** 2022-09-16

**Authors:** Alexander G. Maier, Christiaan van Ooij

**Affiliations:** ^1^ Research School of Biology, The Australian National University, Canberra ACT, Australia; ^2^ Department of Infection Biology, London School of Hygiene & Tropical Medicine, London, United Kingdom

**Keywords:** malaria, cholesterol, host-pathogen interaction, host cell, lipids

## Abstract

Malaria parasites are unicellular eukaryotic pathogens that develop through a complex lifecycle involving two hosts, an anopheline mosquito and a vertebrate host. Throughout this lifecycle, the parasite encounters widely differing conditions and survives in distinct ways, from an intracellular lifestyle in the vertebrate host to exclusively extracellular stages in the mosquito. Although the parasite relies on cholesterol for its growth, the parasite has an ambiguous relationship with cholesterol: cholesterol is required for invasion of host cells by the parasite, including hepatocytes and erythrocytes, and for the development of the parasites in those cells. However, the parasite is unable to produce cholesterol itself and appears to remove cholesterol actively from its own plasma membrane, thereby setting up a cholesterol gradient inside the infected host erythrocyte. Overall a picture emerges in which the parasite relies on host cholesterol and carefully controls its transport. Here, we describe the role of cholesterol at the different lifecycle stages of the parasites.

## Introduction

**Figure 1:**
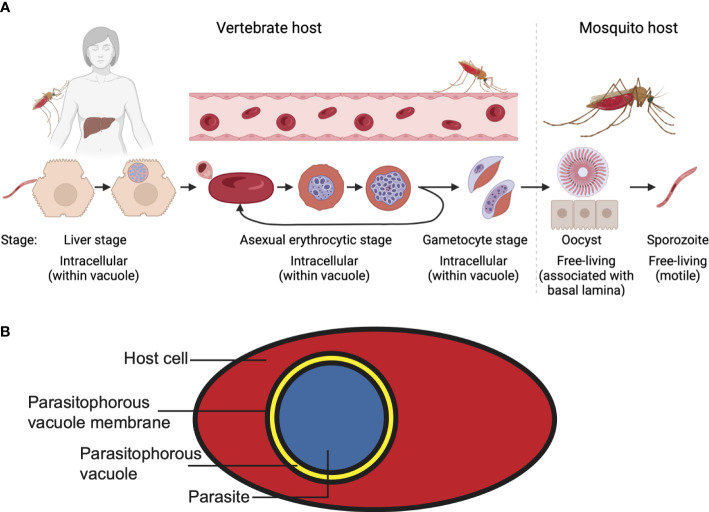
*Plasmodium falciparum* lifecycle. **(A)** For the different stages, the location of the parasite with regards to the host and host cells is indicated. Note the different lifestyles of the parasites. **(B)** A malaria parasite within a parasitophorous vacuole in a host cell. Shown here is an infected erythrocyte.

Malaria parasites replicate in a complex lifecycle involving an *Anopheles* mosquito as the definitive host and a vertebrate as an intermediate host (**Figure 1A**). During this lifecycle, the parasite encounters a wide range of conditions, including different temperatures, nutrient availability, host cells and tissue environments. Infection of the vertebrate host starts when the mosquito injects parasites during a bloodmeal, in the form of sporozoites, into the vertebrate host; these sporozoites then travel through the bloodstream to the liver. There, the parasite invades a hepatocyte by attaching to the host cell plasma membrane and moving itself into the host cell. Subsequently, the parasite is surrounded by a membrane, the parasitophorous vacuole membrane (PVM), that separates it from the cytosol of the host cell. After replicating within the hepatocyte, the progeny (merozoites) is released within a merosome, a membrane-bound structure, that enters the bloodstream, where the merosome lyses, releasing free merozoites. These merozoites subsequently bind and invade erythrocytes, in which they are once again surrounded by a PVM that separates the parasite from the host cell cytoplasm (**Figure 1B**). After growth and asexual replication through schizogony, the erythrocyte bursts to release more merozoites, which in turn bind and invade uninfected erythrocytes. However, a small portion of these parasites commit to sexual development, leading to the formation of female and male gametocytes. These gametocytes are sequestered in the bone marrow, rather than the bloodstream, until they reach a more mature stage of development, at which time they re-enter the bloodstream and can be taken up by mosquitoes during a bloodmeal. In the mosquito gut, the gametocytes differentiate into female and male gametes and subsequently fuse to form a mobile zygote (ookinete), which is free in the midgut. This form of the parasite can traverse the midgut wall, where it forms an oocyst. Although the parasite is not intracellular at the oocyst stage, it is located inside the basal lamina of the midgut. Division of the parasite leads to the formation of sporozoites, which, after release from the oocyst, travel to the salivary glands, from where they are injected into the vertebrate host during the next bloodmeal.

The nature of the cellular environments the parasite encounters during the different life-cycle stages could not be more different: the parasite initially grows as an intracellular parasite in a host cell that can endocytose, divide, produce new proteins and phospholipids and display antigens on its surface (liver stage). In the next lifecycle stage (asexual and sexual erythrocytic stages) the parasite’s host cell does not divide, has no endocytic machinery or membrane biosynthetic capacity. Finally, in the mosquito the parasite converts from a mobile extracellular form (ookinete) to an immobile form (oocyst), which in turn transforms into an extracellular motile stage that can move within the host organism (sporozoite). Furthermore, the parasite grows at vastly different temperatures (35-37°C in the vertebrate host, ambient temperature in the mosquito). These changes in conditions between the lifecycle stages require the parasite to adapt to new conditions at successive lifecycle stages.

Membranes are the immediate contact point between parasites and host cell, hence the properities of both parasite and host membranes are crucial for almost all interactions. One important difference between the host cells and the host tissues that the parasite encounters is the make-up of the different host membranes. The constituents of the host cell membrane determine its biophysical characteristics, such as the stiffness, permeability and fluidity, which can vary widely between cells and organisms (Dufourc, 2008; Ikonen, 2008; Van Meer et al., 2008; Krause and Regen, 2014). Hence, one mechanism by which the parasite responds to the differences between the host environments is by adjusting the composition of host membrane to be more conducive to the parasite’s needs (Hsiao et al., 1991). Furthermore, the different host cells present the parasite with a different pool of lipids it could potentially scavenge and lipid transfer systems that it could take advantage of.

One important regulator of the biophysical properties of a membrane is cholesterol. The importance of cholesterol in the infection of malaria parasites has long been established (Vial et al., 1984; Samuel et al., 2001; Murphy et al., 2007; Murphy et al., 2009) and recent research findings have provided further insight into how cholesterol affects the entry and growth of the parasites. Furthermore, the regulation of cholesterol level in the host cell and the parasite plasma membrane by the parasites has recently come into greater focus. Here we review the current understanding of how cholesterol influences the interaction of the parasite with its host.

## Role of cholesterol in host cell membranes

**Figure 2:**
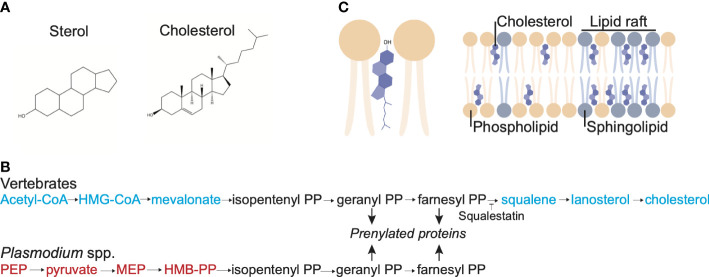
Cholesterol structure, membrane localization and synthesis pathway. **(A)** Structure of the sterol backbone and structure of cholesterol. **(B)** Localization of cholesterol in a lipid bilayer. The sterol backbone of cholesterol intercalates between the acyl groups of the phospholipid in the lipid bilayer and the OH group is associated with the headgroup of the phospholipids. Cholesterol is concentrated in lipid rafts, along with sphingomyelin. **(C)** Synthesis pathway of farnesyl pyrophosphate and cholesterol in vertebrates and *Plasmodium* parasites. The intermediates used in vertebrates but not *Plasmodium* parasites to produce farnesyl pyrophosphate are shown in blue, whereas the intermediates used in *Plasmodium* parasites but not in vertebrates are shown in red; in black are the shared intermediates. Note that *Plasmodium* parasites lack the enzymes required to convert farnesyl PP to cholesterol.

Sterols (including cholesterol) consist of four steroid hydrocarbon rings with a hydroxyl group (**Figure 2A**). In cholesterol and many other sterols, a short *iso*-octyl acyl chain is attached to carbon 17 in one of the hydrocarbon rings (**Figure 2A**). Cholesterol is present in lipid bilayers, with the hydroxyl group nestled between the phosphate groups of the phospholipids in the bilayer and the hydrophobic rings and the hydrocarbon chain positioned between the acyl chains of the phospholipids (**Figure 2B**). Sterols come in many forms along the phylogenetic tree and are often phylum-specific: in vertebrates, the main sterol is cholesterol but sterols are also present in plant cells, mainly in the form of campesterol, sitosterol and stigmasterol, and fungi, as ergosterol (Hannich et al., 2011). Insects depend on the dietary uptake of sterols for membrane functions and hormone production (Behmer and Grebenok, 1998). They lack crucial enzymes for the *de novo* synthesis, however, they have enzymes that allows the conversion of different sterols (Clark and Bloch, 1959).

Cholesterol affects multiple properties of a phospholipid bilayer. The intercalation of the hydrophobic rings of cholesterol between the acyl chains of the phospholipids leads to a condensing effect, decreasing the movement of the acyl chains, thereby decreasing the fluidity and increasing the thickness of the membrane (Holthuis and Menon, 2014). Furthermore, these cholesterol-induced changes in the membrane lead to a decrease in the permeability. However, cholesterol also decreases the temperature at which the membrane transitions to the solid gel state, keeping the membrane fluid at lower temperatures.

In addition to altering the biophysical properties of membranes, cholesterol is also a key force in their functional compartmentalisation. Intercalation of cholesterol can happen through its interaction with various lipids in a membrane. However, owing to the long, saturated acyl chains of sphingomyelin, cholesterol has a particular affinity for sphingomyelin (Kinoshita et al., 2017; Kinoshita et al., 2018). This increased interaction and local aggregation of cholesterol and sphingomyelin in the lipid bilayer results in the formation of detergent-resistant membrane (DRM microdomains [also known as lipid rafts (Coskun and Simons, 2011)]. In turn, certain membrane proteins are enriched in these lipid microdomains, where the spatial constraints and vicinity to other proteins will influence their function. Depending on the nature of the proteins these functions can be related to cell structure, metabolism or signalling. It is worthwhile noting, that the lipid raft paradigm is not without controversy (Sevcsik and Schütz, 2016). However, the general concensus is, that cholesterol aids in the self-organisation of lipid membranes and hence can act as facilitator and stabiliser of protein complexes or as allosteric regulator of protein function (Fantini and Barrantes, 2013).

In vertebrate cells, cholesterol is synthesized in the ER, from where it is distributed throughout the cell. Despite this, the level of cholesterol in the ER is relatively low, with cholesterol levels increasing along the secretory pathway (hence increasing the cholesterol-phospholipids ratio, with concomitant changes in the biophysical properties of the membrane), reaching the highest levels in the plasma membrane (Ikonen, 2008; Van Meer et al., 2008; Holthuis and Menon, 2014). Exchange of cholesterol between organellar membranes is mediated by lipid transfer proteins, which can ferry hydrophilic molecules through aqueous environments by solubilizing cholesterol and then moving through the cytosol (Turner et al., 2012; Wong et al., 2018). Alternatively, cholesterol transfer proteins can act at membrane contact sites, where they promote the exchange the cholesterol between two membranes that are held in close proximity (Mesmin et al., 2019; Garten et al., 2020).

Cells obtain cholesterol from their surroundings, either through the direct uptake of cholesterol from High Density Lipoproteins (HDL) or the internalization and degradation of Low Density Lipoproteins (LDL) particles (Li et al., 2021). After uptake, LDL is degraded in late endocytic organelles and its cholesterol is subsequently transferred from the organelle to other parts of the cell through the action of the proteins LAMP1/2 and NPC1/2 (Eskelinen et al., 2004; Chang et al., 2005; Schneede et al., 2011).

Cholesterol in the plasma membrane is a target for several hemolysins, including Listeriolysin O and Streptolysin O, produced by bacterial pathogens. These hemolysins insert themselves into the membrane through contact with cholesterol and subsequently form a pore. Hemolysins are important tools in cell biology and are also widely used in malaria research for localisation studies or the enrichment of particular lifecycle stages (Ansorge et al., 1997; Brown et al., 2020).

## Synthesis and uptake of cholesterol by malaria parasites

Remarkably, both human host cells that provide a home for the malaria parasite are particularly rich in cholesterol; liver cells are the major synthesis sites for cholesterol in the human body, whereas human red blood cells contain 50% of the circulating cholesterol in humans (Turner et al., 2012). Hence, it is intriguing to speculate about the relationship between host cells and the cholesterol demand of the parasite. Early experiments examining the ability of the parasites to incorporate radiolabeled precursors into cholesterol showed that malaria parasites lack the ability to synthesize cholesterol *de novo* (Trigg, 1968; Cenedella et al., 1969; Lawrence and Cenedella, 1969; Holz, 1977; Sherman, 1979; Maguire and Sherman, 1990; Mbaya et al., 1990; Vial et al., 1990). This finding was confirmed when sequencing of the genome of the malaria parasite *Plasmodium falciparum* revealed that it lacked the genes encoding the enzymes that convert farnesyl pyrophosphate into cholesterol (Carlton et al., 2002; Gardner et al., 2002; Guggisberg et al., 2014) (**Figure 2C**). Phylogenetic investigations point to the presence of sterol synthesis in the earliest eukaryotic organisms, with differences in the synthesis pathway resulting from lineage-specific gene acquisition or gene loss (Desmond and Gribaldo, 2009). The Alveolate lineage, which includes the apicomplexans, lost the genes for sterol synthesis at an early point in evolution (Desmond and Gribaldo, 2009). The parasite nonetheless encodes several orthologues of proteins that may play a role in uptake, binding, transport or modification of cholesterol (**Table 1**). For example, the Fam A protein PF3D7_1463500 (PF14_0604) is a START-domaining protein that has been predicted to bind cholesterol based on the similarity of its predicted structure to that of STARD3 (MLN64), a known cholesterol transport protein (Frech and Chen, 2013). However, the ability of the Fam A protein to bind and transport cholesterol requires experimental validation. Hence, despite the lack of synthesis, the parasite has the potential to interact with cholesterol.

**Table 1:** Putative cholesterol binding proteins of *Plasmodium falciparum*.

Protein	Gene ID (Previous Gene ID)	Expression (a)	Conserved (b)	Essential (c)	Phenotype of mutant (d)	
Fam A	PF3D7_1463500 (PF14_0607)	unknown	All Plasmodium	No (asexual)	ND	(Frech and Chen, 2011; Fougère et al., 2016)
NCR1	PF3D7_0107500 (PFA0375c)	liver stage, asexual (trophozoite, schizont), very low gametocytes, ookinetes, sporozoites	All Plasmodium	Yes (asexual)	lethal	(Istvan et al., 2019)
ABCG2	PF3D7_1426500 (PF14_0244)	female gametocytes (low expression male)	All Plasmodium	No (asexual)	Decreased cholesteryl esters, diacylglycerols and triacylglycerols in gametocytes	(Tran et al., 2014)
Oxysterol binding protein (putative)	PF3D7_1131800 (PF11_0327)	liver stage, asexual stage (trophozoite, schizont), gametocytes (male, female), ookinete	All Plasmodium	No (asexual)	ND	
Phosphatidylcholine-sterol acyltransferase, putative	PF3D7_0629300 (PFF1420w)	liver stage, asexual stage (trophzoite, schizont), oocyst, sprozoite (injected)	All Plasmodium	No (asexual)	Delayed egress from PV in liver stage	(Burda et al., 2015)
PFA66	PF3D7_0113700 (PFA0660w)	asexual stage	Laverania	No (asexual)	Malformation of knobs	(Petersen et al., 2016; Diehl et al., 2021)

Note that in most cases cholesterol binding has not been shown biochemically and is inferred by homology only. ND, Not determined.

The plasma membrane of *P. falciparum*-infected erythrocytes contains less cholesterol compared to uninfected erythrocytes (Maguire and Sherman, 1990; Jackson et al., 2007; Orjih et al., 2008; Hernández-Castañeda et al., 2021). This finding is corroborated by the diminished susceptibility of infected erythrocytes to pore-forming hemolysins and saponin, whose activity is cholesterol-dependent (Jackson et al., 2007; Orjih et al., 2008; Hernández-Castañeda et al., 2021). On the other hand, plasma membranes of murine erthrocytes seemingly do not have such a reduced cholesterol content when infected with rodent *Plasmodium* species (Wunderlich et al., 1991). Whether this difference is a species-specific adaptation or reflects methodological differences remains to be determined.

Furthermore, the parasite plasma membrane contains only very little, if any, cholesterol. This property is exploited for the isolation of intact parasites from erythrocytes using saponin, which lyses membranes in a cholesterol-dependent manner (Christopher and Fulton, 1939; Schlösser, 1969). Several studies have indicated that the phospholipid-cholesterol ratio of isolated parasites is lower than that of uninfected erythrocytes (Rock et al., 1971b; Vial et al., 1984). Notably, these studies used cholesterol-dependent mechanism to isolate parasites from their host cells, which comes with the caveat that saponin also liberates cholesterol from membranes (Böttger and Melzig, 2013) and hence any cholesterol measurements of saponin-isolated parasites are may underestimate the level of cholesterol. Not surprisingly, studies using saponin to isolate *Plasmodium knowlesi* parasites revealed that the cholesterol-phospholipid ratio is lower in the parasite than in the host erythrocyte (Rock et al., 1971a). However, when *Plasmodium chabaudi*-parasites were released from the erythrocytes by osmotic shock, the cholesterol-phospholipid ratio detected in the released parasites was also lower than that of the host erythrocyte (Wunderlich et al., 1991). Overall, the cholesterol-phospholipid ratio was found to be lower in erythrocytes from rats infected with *Plasmodium berghei* compared with erythrocytes from uninfected rats (Lawrence and Cenedella, 1969). A similar result was recently obtained using erythrocytes infected with *P. falciparum* (Hayakawa et al., 2020). The decrease in cholesterol-phospholipid ratio is owing to the significant (4-5-fold) increase of phospholipids found in *Plasmodium*-infected erythrocytes (Lawrence and Cenedella, 1969; Vial et al., 1990). Despite the decreased cholesterol-phospholipid ratio in the infected erythrocytes, the total amount of cholesterol in infected erythrocytes increases compared to uninfected erythrocytes, although to a lesser degree than observed for the phospholipids. This increase was detected *via* diverse biochemical methods and mass-spectroscopy, using different parasite species (Lawrence and Cenedella, 1969; Angus et al., 1971; Botté et al., 2013; Tran et al., 2016; Ridgway et al., 2022). Hence, the infected erythrocyte does seem to take up cholesterol, as even isolated parasites contain cholesterol.

Very little is known about the destination of cholesterol within the infected erythrocyte. Determining the detailed distribution of cholesterol within the parasite-infected host cell is not only hindered by the restrictions of optical resolution but also by the technical limitations of cholesterol-specific probes: some dyes commonly used to detect cholesterol induce changes in environmental conditions (e.g. polarity), rather than detecting cholesterol directly (Klymchenko, 2017). Furthermore, the addition of fluorescent tags alters the physical properties of cholesterol and hence these probes might not behave like untagged cholesterol (Maxfield and Wüstner, 2012). Nonetheless, there is evidence for the uptake of cholesterol by the parasite itself, as cholesterol-specific probes accumulate in asexual parasite and gametocytes (Tran et al., 2016; Hayakawa et al., 2020). Within the parasite, cholesterol is believed to be present in lipid bodies and in the related Apicomplexan parasite *Toxoplasma gondii*, cholesterol has been detected in the rhoptries within the parasite (Jackson et al., 2004; Coppens and Vielemeyer, 2005; Besteiro et al., 2008). Cholesterol was also detected in the parasite plastid (the apicoplast). This is remarkable, as cholesterol is an unusual sterol for plastid membranes (Botté et al., 2013).

As the cholesterol content in infected erythrocyte increases but neither the host erythrocyte nor the parasite has the capacity for *de novo* cholesterol synthesis, it raises the question about the source of cholesterol. Investigations on the delivery of cholesterol to the erythrocyte membrane have been inconclusive: some studies have identified HDL as the external cholesterol source (Grellier et al., 1991), others have identified a transfer of cholesterol from LDL to erythrocytes (Hui et al., 1981; Ohkawa et al., 2020). Advances in detection methods will aid the determination of the exact source and mode of delivery of the cholesterol during the asexual and gametocyte stages.

## Role of cholesterol in invasion and growth of malaria parasites

### Hepatocytes

Cholesterol plays an important role in the invasion and development of the parasite during the liver stage. Treatment of hepatocytes with methyl β-cyclodextran (MβCD), a compound that extracts cholesterol from membranes (Ohtani et al., 1989), decreases the invasion of *P. falciparum* and *Plasmodium yoelii*, but not *P. berghei*, into hepatocytes (Silvie et al., 2006a). Cholesterol may in fact be a limiting factor in the invasion process, as addition of cholesterol to the hepatocyte plasma membrane stimulates parasite invasion (Silvie et al., 2006a). Invasion of *P. falciparum* and *P. yoelii*, but again not *P. berghei*, furthermore requires the hepatocyte surface protein CD81 (Silvie et al., 2003; Silvie et al., 2006a; Silvie et al., 2006b; Silvie et al., 2007). CD81 (being a member of the membrane-spanning tetraspanin family) is present in tetraspanin-enriched membrane domains, which contain proteins and cholesterol (Zuidscherwoude et al., 2015; Robert et al., 2020). CD81 interacts with cholesterol *via* a dedicated cholesterol-binding pocket and bound cholesterol plays an important role in mediating CD81 function (Charrin et al., 2003a; Charrin et al., 2003b; Zimmerman et al., 2016). Anti-CD81 antibodies block invasion of hepatocytes by sporozoites (Silvie et al., 2003; Silvie et al., 2006b; Silvie et al., 2007). However, no parasite ligand for CD81 has been identified and CD81 may not be the actual receptor for the parasite; instead, it may organize the membrane domain that contains the receptor or potential signalling molecules required for parasites invasion (Silvie et al., 2006b).

Once inside the hepatocyte, the parasite is surrounded by a membrane, the parasitophorous vacuole membrane (PVM), that separates it from the host cell cytosol (**Figure 1B**). Staining of hepatocytes infected with *P. berghei* with the cholesterol stain filipin revealed that the parasite is surrounded by a cholesterol-containing membrane (Bano et al., 2007; Petersen et al., 2017). The merosome, which is surrounded by a hepatocyte-derived membrane, also stains brightly with filipin (Sturm et al., 2006; Labaied et al., 2011). The exact function of the cholesterol in the PVM has not been established, but it may have an important function in the regulation of nutrient acquisition. The PVM is permeable to substrates smaller than ~850 Da, allowing the parasite access to nutrients from the host cell (Bano et al., 2007), likely mediated by a pore formed by the parasite protein EXP2 (Gold and Kaplan, 2015; Hakamada et al., 2017; Garten et al., 2018). However, no diffusion of small fluorescent molecules into the parasite was detected after treatment of infected hepatocytes with MβCD, potentially indicating a need for cholesterol for formation or function of the EXP2 pore (Bano et al., 2007).

The PVM expands as the parasite grows, so if the parasite requires a constant level of cholesterol in the PVM, it needs to obtain cholesterol from the host cell. A hepatocyte can acquire cholesterol both through synthesis and uptake. Apart from the *de novo* synthesis in the ER of the hepatocyte (Van Meer et al., 2008; Hannich et al., 2011), Low Density Lipoprotein (LDL) particles containing cholesterylesters are endocytosed and degraded while the cholesterylesters are converted to cholesterol (Brown and Goldstein, 1985). High Density Lipoprotein (HDL), also containing cholesterylesters, are taken up by the hepatocyte *via* the scavenger receptor class B type I (SR-BI)-mediated mechanism (Krieger, 1999; Shen et al., 2017).

In the rodent malaria parasites *P. yoelii* and *P. berghei* it appears that cholesterol from only some of these sources is available to the parasite during the liver stage; when infected hepatocytes are cultured in lipoprotein-deficient serum, the intensity of filipin staining of the parasite is less than in infected hepatocytes cultured in the presence of LDL, indicating that the parasite can obtain cholesterol from LDL (Labaied et al., 2011). Furthermore, fluorescent cholesterol accumulates in the PVM when hepatocytes are incubated in the presence of LDL containing fluorescently labeled cholesterol (Labaied et al., 2011). Interestingly, no such accumulation was detected when fluorescently labeled cholesterol HDL particles were added, indicating that only the LDL pathway supplies cholesterol to the parasite from the medium at this stage (Labaied et al., 2011). This is consistent with the notion that HDL is responsible for the removal of excess cholesterol from the body. Furthermore, radiolabel was detected in merosomes released from hepatocytes incubated in the presence of radiolabeled cholesterol, further indicating that cholesterol added externally to the host hepatocyte can be incorporated into the PVM or parasites themselves (Labaied et al., 2011).

The parasite also directly obtains cholesterol synthesized *de novo* by the hepatocyte. Incubation of the infected hepatocytes with radiolabeled HMG-CoA, a precursor in the cholesterol synthesis pathway (**Figure 2C**), leads to the incorporation of radiolabeled cholesterol in the released merosome (Labaied et al., 2011). Furthermore, inhibition of *de novo* cholesterol synthesis by the hepatocyte using squalestatin, which inhibits squalene synthase (**Figure 2C**), decreases the cholesterol content of the parasite, although it does not affect the growth of the parasites (Labaied et al., 2011). As *P. berghei* mutants lacking the protein UIS4 take up less filipin, this parasite protein may play a role in the uptake of cholesterol (Petersen et al., 2017).

Together, these experiments reveal that the parasite actively acquires cholesterol from the host hepatocyte, by intercepting cholesterol taken up through LDL and cholesterol synthesized *de novo*. Different studies that have investigated the effect of lowering the cholesterol levels in the host hepatocyte obtained different results. In some cases no effect on parasite growth was detected (Labaied et al., 2011). This was the case when infected hepatocytes were grown in lipoprotein-depleted medium, when LDL receptor levels were decreased using siRNA or when squalene synthase activity level was decreased using either an inhibitor or siRNA (Labaied et al., 2011). In contrast, the growth of the parasite was affected and fewer progeny were produced when the transfer of cholesterol from the lysosome was prevented, either by genetically reducing the levels of the lysosomal proteins LAMP2 or NPC1/2 or through the addition of U18666A, a compound that blocks the exit of LDL-derived free cholesterol from late endosomes of the host (Petersen et al., 2017).

The difference in the results may reflect the amount by which cholesterol acquisition by the parasites was affected and indicate that under normal circumstance cholesterol availability is not a rate-limiting step for optimal parasite replication. In support of this, supplementing the medium of infected hepatocytes with additional LDL did not increase the production of progeny (Labaied et al., 2011). The function of the cholesterol in the growth of the parasite is not known; potentially metabolic functions, including the function of the EXP2 pore, require cholesterol (Bano et al., 2007). Nonetheless, cholesterol clearly plays an essential role for invasion replication of the parasite in hepatocytes, although the exact function during these steps remains to be identified.

### Erythrocytes

#### Asexual stage

Similar to invasion of hepatocytes, invasion of erythrocytes requires cholesterol in the host cell membrane. The cholesterol-phospholipid ratio of animal cells lies normally between 1:1 and 0.8:1 (Van Meer et al., 2008), indicative of a functional significance of the higher cholesterol content. This ratio is independent of the level of cholesterol in the surrounding serum, as erythrocytes from people or mice with high serum cholesterol levels maintain a 1:1 cholesterol-phospholipid ratio (Koch et al., 2019). Artifically reducing the cholesterol level in erythrocytes by treatment with MβCD results in fewer parasites being able to invade these cells (Dluzewski et al., 1985; Samuel et al., 2001; Murphy et al., 2009; Koch et al., 2019; Geoghegan et al., 2021; Ahiya et al., 2022). Parasites trying to invade the MβCD-treated cells follow through with the early stages of the invasion, forming a tight interaction with the cell and induce a Ca^2+^ flux, but ultimately are unable to complete invasion (Koch et al., 2019; Ahiya et al., 2022). Hence, cholesterol appears to be required at a later stage of the invasion process (Weiss et al., 2015; Geoghegan et al., 2021). In contrast to the invasion of hepatocytes, artificially increasing the cholesterol level in erythrocytes has little effect on the invasion efficiency of the parasite and invasion efficiency actually decreases at high cholesterol concentrations (Dluzewski et al., 1985; Koch et al., 2019). The exact effect of the cholesterol depletion on invasion is not known; it is likely that the disruption of lipid rafts prevents the parasites from engaging with an essential receptor or leads to loss of lipid raft-based signaling (Samuel et al., 2001; Harrison et al., 2003). Supporting the notion that the effect of the MβCD treatment is caused by the disruption of rafts is the finding that treatment of erythrocytes with lidocaine, which disrupts rafts (Kamata et al., 2008), also prevents parasite invasion (Koshino and Takakuwa, 2009). Furthermore, it has been suggested that cyclosporin mediates its antimalarial properties through its effect on lipid rafts (Azouzi et al., 2011).

Finally, inside the erythrocytes, the parasites are – like in the hepatocyte – encased by a PVM, which also contains host cell-derived cholesterol. This cholesterol has been introduced during the invagination of the erythrocyte membrane in the course of the invasion process (Tokumasu et al., 2014; Geoghegan et al., 2021). Measurement of the cholesterol concentration in the PVM immediately after completion of invasion indicated that cholesterol is concentrated in the PVM, leading to a decrease in the cholesterol concentration in the erythrocyte plasma membrane (Geoghegan et al., 2021). This finding confirmed other studies, either investigating purified erythrocyte lipids or using cholesterol-sensitive probes, that showed that the cholesterol level in the infected erythrocyte plasma membrane was reduced after parasite invasion and revealed the presence of cholesterol derived from the erythrocyte membrane in the PVM (Maguire and Sherman, 1990; Tokumasu et al., 2014; Fraser et al., 2021).

The decrease of cholesterol in the infected erythrocyte membrane has important consequences for the evasion of the host immune system. The enzymes flippase and scramblase, which regulate the lipid asymmetry across the membrane leaflets of the erythrocyte membrane, are sensitive to cholesterol level in the surrounding membrane (Van Zwieten et al., 2012; Tokumasu et al., 2014; Arashiki et al., 2016; Hayakawa et al., 2020; Fraser et al., 2021). Hence, a decrease in the level of cholesterol affects the lipid asymmetry in the plasma membrane, leading to an increased exposure of phosphatidylserine and phosphatidylethanolamine in the outer leaflet (Joshi et al., 1987; Fraser et al., 2021). The surface exposure of phosphatidylserine in particular leads to increased phagocytosis of infected erythrocytes by monocytes and hence the parasites has to invest additional energy to maintain higher flippase activity (Fraser et al., 2021). In addition, the altered biophysical properties of the infected erythrocytes membrane caused by cholesterol reduction might also influence further parasite-host interactions, including the fluidity of the erythrocyte plasma membrane and the formation of lipid rafts, which may have a bearing on the ability of the spleen to filter out infected erythrocytes.

Cholesterol depletion increases the fluidity of the inner plasma membrane leaflet (Chabanel et al., 1985). Even after invasion, the parasite requires that the cholesterol concentration in the erythrocyte plasma membrane remains above a certain threshold, as removing cholesterol from the plasma membrane of infected erythrocytes using MβCD leads to rapid extrusion of the parasite. The extracellular parasite remains surrounded by the PVM, albeit a broken one (Lauer et al., 2000; Samuel et al., 2001; Ahiya et al., 2022) and seems to remains viable for about 24 hours (Lauer et al., 2000; Samuel et al., 2001; Ahiya et al., 2022). The parasites do not enter schizogeny and hence do not replicate further (Samuel et al., 2001). Interestingly, the erythrocyte itself does not disintegrate during the extrusion of the parasite (Samuel et al., 2001; Ahiya et al., 2022), although the MβCD treatment increases the permeability of the plasma membrane sufficiently to allow phalloidin to enter the erythrocyte (Ahiya et al., 2022). The mechanism of extrusion is unclear; it is very rapid, occurring over a span of about ten seconds and is insensitive to the microtubule inhibitor cytochalasin D, indicating that the extrusion is not powered by the parasite, since cytochalasin D interferes with the parasite actin cytoskeleton. Inhibitors of the parasite proteins ATP4 or NCR1, which increase the cholesterol level in the parasite plasma membrane, decrease the effect of the removal of cholesterol from the erythrocyte, although the reason for this remains unclear (Das et al., 2016; Bhatnagar et al., 2019). The extrusion of parasites has only been observed with trophozoites; ring-stage parasites remain in the erythrocyte upon MβCD-treatment. However, ring-stage parasites in MβCD-treated erythrocytes do not develop properly, forming oddly shaped schizonts, and produce fewer progeny, indicating that the cholesterol level in the plasma membrane of the host cell remains important for the development of the parasite (Frankland et al., 2006; Ahiya et al., 2022). Despite the lowered cholesterol level in the erythrocyte plasma membrane, parasites treated during the ring stage are not extruded when they mature to the trophozoite stage (Ahiya et al., 2022), potentially indicating that the parasite can adapt and redistribute cholesterol in the ring stage to prevent extrusion when it reaches the trophozoite stage.

Furthermore, cholesterol in the erythrocyte plasma membrane is required at later intraerythrocytic stages for the display of parasite proteins on the cell surface. Certain parasite proteins (or the machinery required for transporting these proteins) have an affinity for lipid rafts in the host cell membrane – disruption of lipid rafts through the treatment of infected cells with MβCD prevents transport of the parasite protein PfEMP1 to the surface of the infected cell (Frankland et al., 2006). It is likely that the knobs on the surface of the infected erythrocyte represent cholesterol-rich membrane rafts, as these structures are Triton X-100-insoluble but are solubilized in 2% SDS, the same conditions that enrich membrane rafts (Schravendijk et al., 1993).

**Figure 3:**
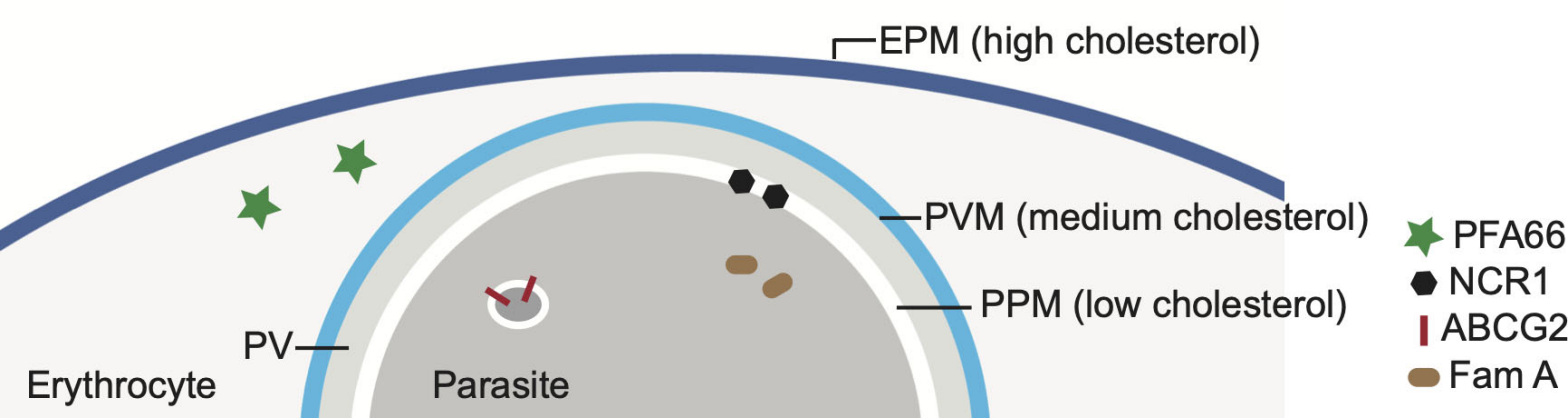
Cholesterol gradient in erythrocytes infected with *Plasmodium falciparum* and localization of several putative cholesterol-binding or transport proteins. The level of cholesterol in each membrane – the erythrocyte plasma membrane (EPM), the parasitophorous membrane (PVM) and the parasite plasma membrane (PPM) – is indicated by the blue coloring. The EPM contains the highest concentration of cholesterol, followed by the PVM and then the PPM. PV-parasitophorous vacuole. Also indicated are the locations of several parasite proteins that are known or have been proposed to have a role in cholesterol transport. See **Table 1** for more information about these proteins.

Detailed investigation of the distribution of cholesterol in the infected erythrocyte revealed the presence of an inward cholesterol gradient: the highest level of cholesterol is found in the erythrocyte plasma membrane, a lower level in the PVM and even less cholesterol in the parasite plasma membrane (Tokumasu et al., 2014; Hayakawa et al., 2020) (**Figure 3**). It appears the parasite actively maintains this gradient, in part by removing cholesterol from the parasite plasma membrane. For this it uses an orthologue of the Niemann-Pick Type C protein, NCR1 (PF3D7_0107500), the human orthologue of which, NPC1, binds cholesterol, whereas the *Saccharomyces cerevisiae* orthologue binds sphingomyelin (Malathi et al., 2004). NCR1 is present in the parasite plasma membrane and is essential for the parasite – inhibition or genetic knock-down leads to changes in digestive vacuole formation and, interestingly, makes the parasite itself sensitive to saponin, indicating that cholesterol accumulates in its plasma membrane (Istvan et al., 2019). Hence, NCR1 may be responsible for removing the cholesterol that the parasite takes up when it ingests hemoglobin – and takes up part of the cholesterol-containing PVM in the process – through the cytostome from the parasite plasma membrane. Similarly, Inhibition of ATP4 leads to accumulation of cholesterol within the parasites, with similarly lethal consequences (Bhatnagar et al., 2019). How the accumulation of cholesterol inside the parasite leads to the phenotypes that are detected remains unclear, however.

Although the PVM contains a lower level of cholesterol than the erythrocyte plasma membrane, the PVM does appear to contain DRMs; the parasite protein EXP1 and the *Plasmodium* Translocon of Exported Proteins (PTEX) are both located in the PVM and are present in DRMs (Lauer et al., 2000; Samuel et al., 2001; Murphy et al., 2007; de Koning-Ward et al., 2009). The presence of DRMs in the PVM raises the possibility that the spatial separation detected between PVM proteins, such as certain ETRAMPs and EXP1 (Spielmann et al., 2003; Spielmann et al., 2006), reflects different affinities of these proteins for DRMs. However, it should be noted that the presence of ETRAMPS in DRMs has not yet been established, and hence the spatial separation may also be determined by other factors.

Cholesterol is also present in the cytosol of infected erythrocytes, as part of J-dots. These are small proteinaceous structures containing the heat shock protein PfHSP70x and the J-domain proteins PFA66 and PFE55 (Külzer et al., 2010; Petersen et al., 2016; Diehl et al., 2021) and may play a role in the transport of transmembrane-domain containing proteins, including PfEMP1, through the cytoplasm of the host cell. PFA66 – important for the proper formation of knobs – binds cholesterol directly (Behl et al., 2019; Diehl et al., 2021). This interaction could potentially serve to transport cholesterol through the erythrocyte cytosol, be involved in the tethering of J-dots to the erythrocyte plasma membrane or act as a stabilizer for the transmembrane proteins transported by the J-dots. Erythrocytes infected with mutant parasites expressing a version of PFA66 that lacks the putative cholesterol binding region at its C terminus contain aberrant, greatly extended knobs (Diehl et al., 2021).

These findings demonstrate that cholesterol plays multiple roles in the growth of the parasite in erythrocytes, from its requirement for invasion to the stabilization of the infected erythrocyte to the dependence of the parasite on cholesterol for the formation of functional knobs.

#### Gametocytes

The role of cholesterol in gametocytes has been explored less than in asexual stages, despite the fact that the amount of cholesterol in gametocyte-infected erythrocytes increases significantly during maturation (Tran et al., 2016). There are three potential reasons for such an increase in cholesterol: regulation of deformability, functionalisation of membranes and storage for use upon transmission to the mosquito.

Early gametocyte stages are stiff and cytoadhere in the bone marrow (Rogers et al., 2000) (presumably as a form of immune-evasion) (Rogers et al., 2000). Mature gametocytes on the other hand are deformable and circulate in the blood of the human host, which allows for passage through the spleen (Aingaran et al., 2012; Duez et al., 2015; Dearnley et al., 2016) and distribution to the periphery of the human body to ensure efficient transmission to mosquitoes. The molecular mechanism underlying this shift in deformability most certainly involves both a change in lipid composition (including cholesterol) and the rearrangement of the inner membrane complex of gametocytes (Dixon et al., 2012; Schneider et al., 2017). Another regulator of membrane fluidity, sphingomyelin, also increases during maturation (Tran et al., 2016), consistent with the notion that the observed changes in deformability of the gametocyte-infected erythrocyte during the sexual development are also driven by membrane lipid composition. Notably, male and female gametocytes contain a similar amount of cholesterol (Ridgway et al., 2022).

A study that analysed the proteome of the DRM microdomains of gametocytes (Fratini et al., 2017) assigned an involvement of these domains in contact and exchange sites between organelles (e.g. ER and mitochondria) or secretory vesicles (Lahiri et al., 2014), attachment sites for chromatin (Albi et al., 2013) or transcription processes at the nuclear membrane based on the nature of the proteins identified (Cascianelli et al., 2008).

As the transition from the vertebrate host to the mosquito also involves a switch from a high-cholesterol to a low-cholesterol environment (see below), the parasite might stockpile cholesterol during the gametocyte development in the human host. Excess free cholesterol is toxic to cells and results in decreased membrane fluidity, disrupted membrane microdomains and altered membrane protein function (Musso et al., 2013). Hence, many cells convert and store excess hydrophobic cholesterol in the form of more water-soluble cholesteryl esters (CE) (Brown et al., 1980). Whereas erythrocytes infected by asexual parasites contain very little CE, the level of CE in the erythrocyte increases more than 6-fold during gametocyte maturation (Tran et al., 2016). This points to a role of CE in transmission, which is further corroborated by the finding that female gametocytes contain over 4 times more CE than male gametocytes (Ridgway et al., 2022). Since female gametocytes provide the cell body for the future oocyst, whereas the contribution of the male gametocyte is restricted to delivery of DNA, female-specific accumulation of nutrients to be carried over to the mosquito seems logical.

### Mosquito stages

The parasite’s access to cholesterol in the mosquito is more restricted than in the human host. Cholesterol is essential for the biological function of insect cellular membranes, and without cholesterol insects cannot complete their life cycle (Canavoso et al., 2001). However, similar to human erythrocytes, mosquitoes are unable to synthesise cholesterol, as they lack key enzymes of the cholesterol synthesis pathway. Hence, mosquitoes have to obtain cholesterol from their diet (Clark and Bloch, 1959; Canavoso et al., 2001). Apart from being used for their biological membranes, mosquitos use cholesterol to synthesise ecdysteroids, hormones important for their egg development (Clifton and Noriega, 2012; Talyuli et al., 2015).

Since the parasite and mosquito both rely on the uptake of cholesterol, once inside the mosquito midgut, the parasite very quickly faces a low cholesterol environment. One would therefore expect the parasite to possess mechanisms by which it can overcome this bottleneck (for example, by accumulating cholesterol or CE in the gametocyte stages where cholesterol can be easily obtained and storing it for use in the insect phase of the life cycle). There are precedents for the impact of resource restriction on disease transmission in mosquitoes: mosquito cells infected with *Wolbachia* bacteria display aberrant intracellular cholesterol trafficking and localised cholesterol accumulation. This in turn renders the cell more resistant to viral infection (Geoghegan et al., 2017). A mosquito diet particular rich in cholesterol on the other hand, makes the insects more susceptible to viral infection (Caragata et al., 2013). It has been shown previously that *Wolbachia* infection of *Anopheles stephensi* mosquitos renders the mosquitos refractory to infection with *Plasmodium* parasites (Bian et al., 2013). Although it was speculated that reactive oxygen species produced by the bacteria inhibite the parasites, it would be interesting to explore whether availability of cholesterol might also play a role in the modulation of mosquito susceptibility to infection with *Plasmodium* parasites.

## Conclusion

During the life-cycle transition the parasite encounters various environments with different cholesterol availabilities. Hence the parasite has developed various strategies to ensure that enough cholesterol is available to cover the significant demand that is fuelled by the massive proliferation and consequent membrane synthesis of the parasite. The detailed analysis of the way the malaria parasites acquires and utilises cholesterol might identify opportunities to interfere with *Plasmodium* infections.

As outlined above, cholesterol is important for the invasion of host cells in the human host. Cholesterol is also crucial for the correct development of parasite induced structures in the host erythrocyte (e.g. PVM and knobs) and the correct transport of parasite molecules important for host-parasite interactions (e.g. PfEMP1). It further impacts host-parasite interactions by modulating the activity of some transmembrane proteins (e.g. flippase) or the deformability of infected erythrocytes. It is very likely that in time additional functions of cholesterol important for the survival of the parasite will be uncovered and added to this already impressive list.

Although significant findings have been made in the last few years, there are still many open questions: e.g., what is the precise mechanism of cholesterol uptake? How is cholesterol distributed within the infected erythrocyte, parasite and within the various membranes? Are there differences in the different *Plasmodium* species, and if yes, do they reflect host-specific adaptations? How exactly does the parasite utilise and store cholesterol?

Without a doubt cholesterol plays a key role in the interplay between host and parasite, however, it remains to be seen whether the underlying infrastructure that allows the parasite to exploit the host cholesterol provides distinctive features for interference.

## Author contributions

AM and CvO wrote and edited this manuscript. All authors contributed to the article and approved the submitted version.

## Acknowledgments

AM is supported by the Australian National Health and Medical Research Council (GNT1182369) and the Australian Research Council (DP1801032), CvO is supported by the Medical Research Council Career Development Award MR/R008485/1. **Figures 1**, **2** were produced in part using BioRender.com and ChemDraw.

## Conflict of interest

The authors declare that the research was conducted in the absence of any commercial or financial relationships that could be construed as a potential conflict of interest.

## Publisher’s note

All claims expressed in this article are solely those of the authors and do not necessarily represent those of their affiliated organizations, or those of the publisher, the editors and the reviewers. Any product that may be evaluated in this article, or claim that may be made by its manufacturer, is not guaranteed or endorsed by the publisher.
